# Clinical and Multi-Mode Imaging Features of Eyes With Peripapillary Hyperreflective Ovoid Mass-Like Structures

**DOI:** 10.3389/fmed.2022.796667

**Published:** 2022-02-09

**Authors:** Xiao Xie, Tingting Liu, Wenqi Wang, Ge Tian, Jinyan Wang, Jitian Guan, Meng Chen, Xunchang Wang, Qingjun Zhou

**Affiliations:** ^1^The First Clinical Medical College of Shandong University of Traditional Chinese Medicine, Jinan, China; ^2^Eye Hospital of Shandong First Medical University (Shandong Eye Hospital), Jinan, China; ^3^State Key Laboratory Cultivation Base, Shandong Provincial Key Laboratory of Ophthalmology, Shandong Eye Institute, Qingdao, China; ^4^Shandong First Medical University and Shandong Academy of Medical Sciences, Qingdao, China; ^5^School of Ophthalmology, Shandong First Medical University, Jinan, China; ^6^Zaozhuang Shizhong District People's Hospital, Zaozhuang, China; ^7^Qingdao Eye Hospital of Shandong First Medical University, Qingdao, China

**Keywords:** peripapillary hyperreflective ovoid mass-like structures, optic disc drusen, optic disc edema, optical coherence tomography, regional blood flow imaging, multi-mode imaging features

## Abstract

**Purpose:**

To observe and analyze the clinical and multi-mode imaging features of eyes with PHOMS, and to introduce two cases of PHOMS which underwent multi-mode imaging.

**Methods:**

Retrospective clinical observational study. A total of 26 patients (37 eyes) with hyperreflective structures surrounded by hyporeflective edges around the optic discs who were examined and diagnosed at Shandong Eye Hospital between January 2019 and June 2021 were included in the study. Among these patients, 12 were male and 14 were female. Fifteen were monocular. The average age was 39 years. All patients underwent the following examinations: Best-corrected visual acuity (BCVA), intraocular pressure examinations, slit-lamp anterior segment examinations, indirect ophthalmoscopy, visual field examinations, fundus color photography, fundus autofluorescence (FAF), optical coherence tomography (OCT), and optical coherence tomography angiography (OCTA). Some of the patients were examined with fundus fluorescein angiography (FFA). Clinical data and imaging characteristics from the OCT, OCTA, and FFA were analyzed retrospectively.

**Results:**

We found the hyperreflective structures surrounded by hyporeflective edges around the optic discs in 37 eyes. EDI-OCT results revealed hyperreflective structures surrounded by hyporeflective edges around the optic discs in all eyes. Typical hyperreflexia lesions occurred around the optic disc, located subretinally and above Bruch's membrane. OCTA revealed that the highly reflective perioptic material also had vascular structures.

**Conclusion:**

EDI-OCT of PHOMS showed hyperreflective structures surrounded by hyporeflective edges around all of the optic discs. Infra-red photography showed temporal hyperreflexia. These characteristics can be seen in a variety of diseases and may be a relatively common feature revealed by EDI-OCT scanning. These characteristics may also be seen in elderly patients as well as children. PHOMS may be found in optic disc drusen (ODD), tilted disc syndrome (TDS), optic neuritis, ischemic optic neuropathy, and in white dot syndromes. Few patients may be developed into macular neovascularization (MNV). In order to improve the accuracy and robustness of the conclusions and provide better clinical guidance, we need to conduct more comprehensive research in the subsequent clinical work.

## Introduction

Peripapillary hyperreflective ovoid mass-like structures (PHOMS) are new imaging feature that have recently been revealed by EDI-OCT. In previously published literature, PHOMS was often diagnosed as buried optic disc drusen (ODD). It is also easily confused with optic disc edema (ODE) (1–7). In 2018, the term “PHOMS” was proposed by the Optic Disc Drusen Studies (ODDS) consortium to characterize the hyperreflective ovoid mass-like structures found on OCT images in the areas around the optic disc (8). The pathogenesis of PHOMS is not clear. Most researchers think it is a herniation of nerve fibers or axoplasmic stasis, or is related to congestion in the prelaminar optic nerve head (9, 10). Understandings of PHOMS have improved because of the application of optical coherence tomography (OCT), enhanced depth imaging optical coherence tomography (EDI-OCT) and optical coherence tomography angiography (OCTA). We recommend adopting this refined definition (11). Although there have been some reports about this disease, there are few studies about the clinical features of PHOMS that are based on multi-modal imaging. Here, we retrospectively analyzed the clinical manifestations and multi-modal imaging features of eyes with PHOMS. The OCT finding was investigated in a larger, more definitive, series of patients and termed PHOMS, and we found that PHOMS existed in some optic nerve diseases. This may indicate that PHOMS are novel and not well-characterized OCT findings occurring in several disorders of the optic nerve. We expect to provide more evidence for the clinical diagnosis and treatment of the disease.

## Methods

This was a retrospective clinical observational study. The study was approved by the Institutional Ethics Committee of Shandong eye hospital (approval No. 2019-G-012) on March 12, 2019. All experimental procedures were conducted in accordance with the Declaration of Helsinki (World Medical Association, as amended 2013) and the Health Insurance Portability and Accountability Act. All of the patients in this study agreed to provide identification information that was relevant to their pathology, and signed informed consent as required. A total of 26 patients (37 eyes) with PHOMS who were examined and diagnosed at Shandong Eye Hospital between January 2019 and June 2021 were included in the study. Among these patients, 12 were male and 14 were female. Fifteen were monocular. The average age was 39 years. The time from symptom onset to treatment ranged from 1 to 45 days, with an average time of 11 days. All patients complained of vision loss.

### Inclusion Criteria

(1) The rim of the optic disc was blurred and bleeding could be seen in fundus color photography; (2) EDI-OCT revealed a hyperreflective structure surrounded by a hyporeflective edge around the optic disc.

### Exclusion Criteria

(1) The ocular fundus was affected by severe refractive stroma opacity; (2) After repeated measurements, it was still not possible to obtain images that meet the analysis requirements.

All patients underwent the following examinations: Best-corrected visual acuity (BCVA) [with the international standard chart of vision and the minimum resolution logarithmic (LogMAR) vision chart], intraocular pressure examinations, slit-lamp examinations, indirect ophthalmoscopy, visual field examinations, fundus color photography, fundus autofluorescence (FAF), optical coherence tomography (OCT), and optical coherence tomography angiography (OCTA). Some of the patients were examined with fundus fluorescein angiography (FFA). We retrospectively analyzed the patients' clinical data OCT, OCTA, and FFA imaging characteristics.

### OCT, OCTA, and FFA

We selected Heidelberg Spectralis HRA+OCT (Heidelberg Engineering, Heidelberg, Germany) for these examinations. The IR mode was used to track the subjects' optic discs, followed by the appropriate mode was used to obtain the structural images or retinal blood flow of the optic discs. When patients underwent FFA examinations, their skin tests were negative, and 3 ml of 20% fluorescein sodium was quickly injected into the middle elbow vein within a 5 s window.

Each patients' examinations were performed by the same doctor on the same day. If the position and quality of the image were not satisfactory, or if there were too many motion artifacts, the images were recorded again. The resulting images were reviewed by two other ophthalmologists with professional training. When there was a discrepancy between the ophthalmologists, a third ophthalmologist reviewed the scans so they could all reach a consensus. After the diagnosis, the primary disease treatment principle carried through intervention treatment, and patients received regular follow-up care.

## Results

### Basic Characteristics of Patients

We found the hyperreflective structures surrounded by hyporeflective edges around the optic discs in 26 patients (37 eyes). As shown in Table 1, Among these patients, 12 were male and 14 were female. Fifteen were monocular. The average age was 38.65 ± 18.76 (Mean ± SD) years. The average BCVA was 0.55 ± 0.38 (Mean ± SD). PHOMS was present in patients diagnosed with a variety of diseases, including optic disc drusen (nine eyes), tilted disc syndrome (two eyes), optic neuritis (11 eyes), ischemic optic neuropathy (12 eyes), white dot syndromes (two eyes) and macular neovascularization (one eye). PHOMS widths ranged from 309 μm to 2285 μm (Mean ± SD: 721.14 ± 365.11), and their heights ranged from 194 μm to 839 μm (Mean ± SD: 399.30 ± 145.98).

**Table 1:** Basic patient characteristics.

**Number**	**Sex**	**Age**	**Eye**	BCVA^*^(LogMAR)	**PHOMS** ^*^	
					**Width (μm)**	**Height (μm)**
1	Male	54	OD	0.1	1427	504
2	Male	41	OS	1.0	619	312
3	Male	55	OD	0.7	678	402
			OS	0.4	602	377
4	Male	45	OD	0.3	560	386
			OS	0.1	745	333
5	Male	48	OD	0.2	729	225
6	Male	20	OD	0.3	555	454
			OS	0.3	757	640
7	Male	67	OD	0.2	898	547
8	Male	33	OD	0.8	485	321
			OS	0.9	530	517
9	Male	55	OD	1.0	685	503
			OS	0.9	342	279
10	Male	48	OD	0.1	845	306
			OS	0.1	793	452
11	Male	55	OD	1.0	347	247
			OS	0	309	256
12	Male	66	OS	0.2	855	404
13	Female	9	OD	0	2285	798
			OS	0.5	1604	839
14	Female	13	OS	0.9	424	427
15	Female	61	OD	0.7	464	219
			OS	1.0	598	301
16	Female	14	OD	1.0	625	286
			OS	0.9	596	372
17	Female	14	OD	0.3	766	392
18	Female	36	OS	1.0	556	243
19	Female	44	OD	0.9	722	365
20	Female	9	OS	0.7	952	490
21	Female	24	OS	1.0	492	413
22	Female	18	OD	1.0	512	595
23	Female	64	OS	0.4	693	378
24	Female	54	OD	1.0	506	319
25	Female	29	OD	0	471	194
			OS	0	875	422
26	Female	29	OD	0.6	780	256

* *BCVA, best-corrected visual acuity; PHOMS, peripapillary hyperreflective ovoid mass-like structures; OD, oculus dexter; OS, oculus sinister*.

### Cases Sharing

Below, we have selected two cases with different characteristics to introduce PHOMS in detail.

#### Case 1

**Figure 1:**
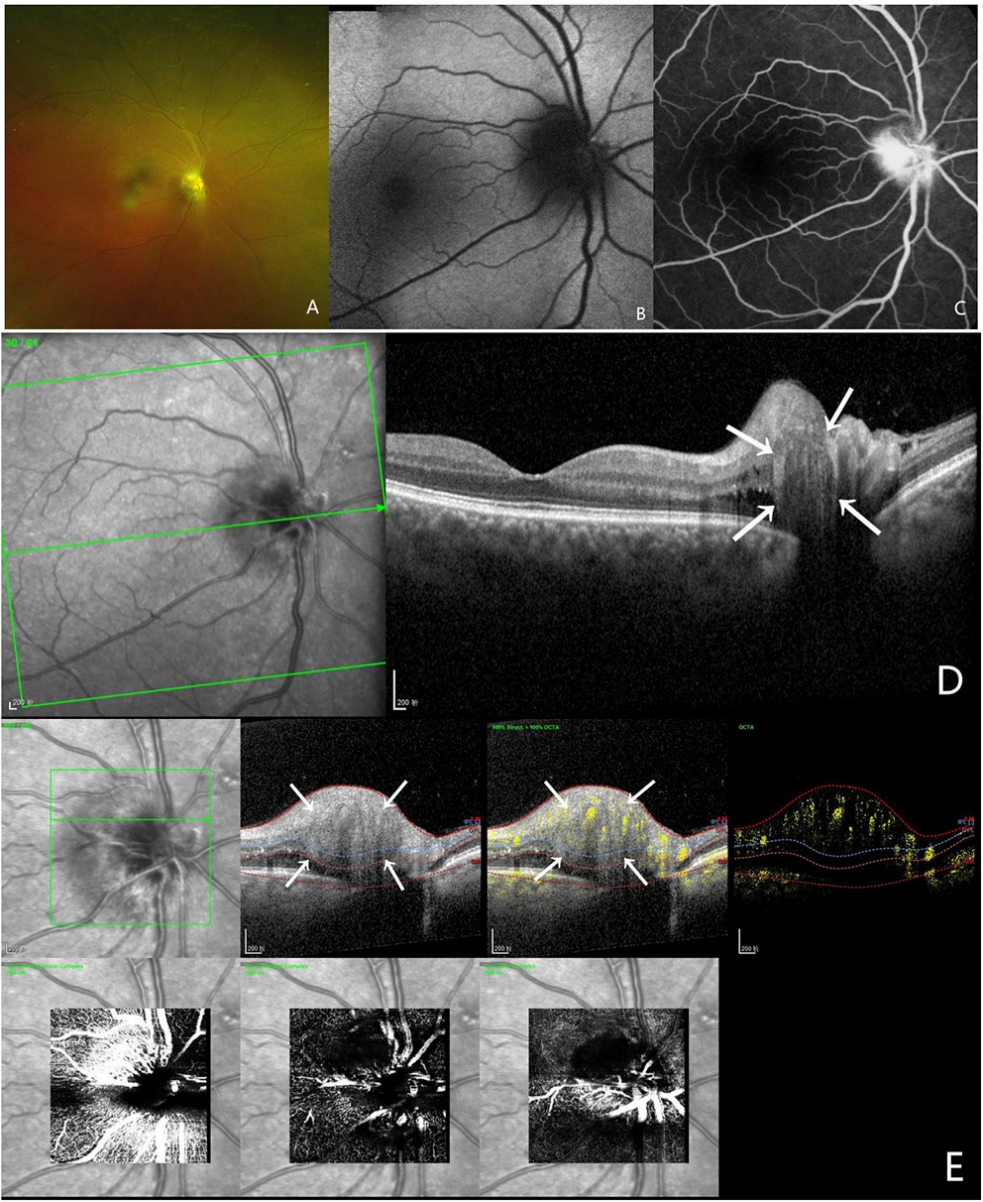
Multi-modal image data of case 1. **(A)** Color fundus photography showed blurred boundaries and bleeding of the optic disc. **(B)** This case did not show autofluorescence. **(C)** FFA shows a hyperreflective optic disc with poorly defined boundaries. **(D)** OCT confirmed the presence of PHOMS, which is a hyperreflective ovoid mass-like structure surrounded by a hyporeflective edge around the optic disc. **(E)** OCTA showed a vascular complex, and there was a strong blood flow signal within this hyperreflective lesion.

A 54-year-old man came to our department complaining of reduced visual acuity in the right eye. LogMAR BCVA was 0.1 for right eye. Intraocular pressure and slit-lamp anterior segment examinations were normal. The initial diagnosis was ischemic optic neuropathy in the right eye (Figure 1).

#### Case 2

**Figure 2:**
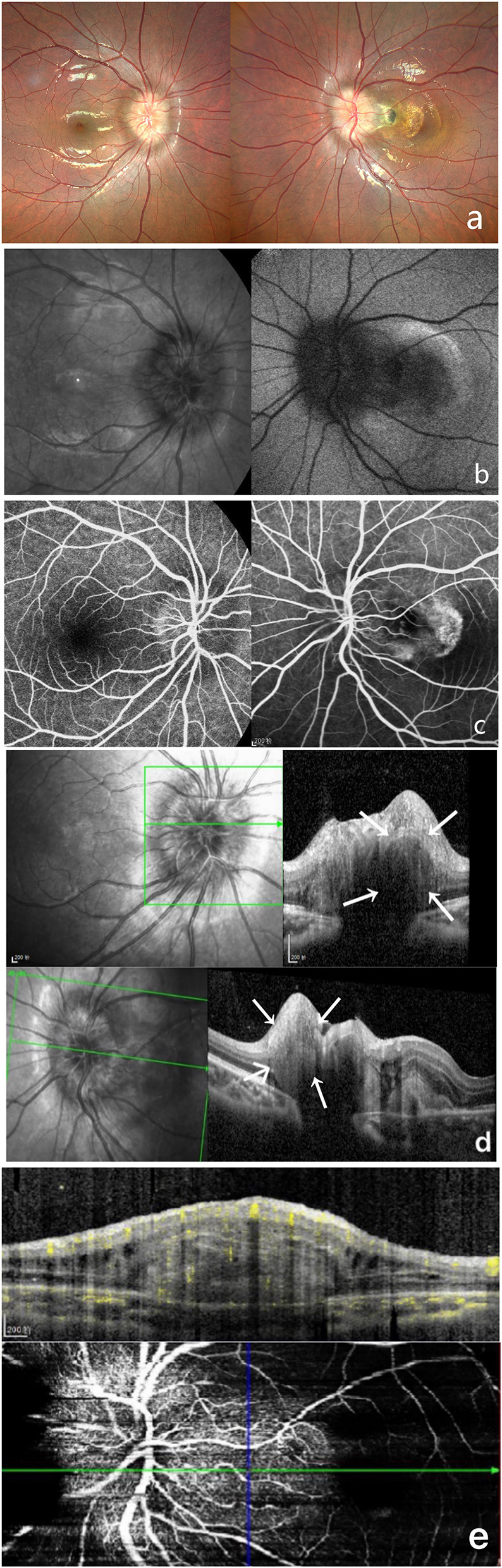
Multi-modal image data of case 2. **(a)** Color fundus photography showed unclear optic disc boundaries, as well as are fibrosis and serous detachment of the retina in macular area. **(b)** This case did not show autofluorescence. **(c)** FFA shows a hyperreflective optic disc with poorly defined boundaries. **(d)** OCT shows a hyperreflective ovoid mass-like structure surrounded by a hyporeflective edge around the optic disc. **(e)** OCTA shows blood flow within this hyperreflective lesion.

A 9-year-old girl was brought to our department by her mother complaining of reduced visual acuity in the left eye. LogMAR BCVA examination was 0 for the right eye and 0.5 for the left eye. Intraocular pressure and slit-lamp anterior segment examinations were normal. The initial diagnosis was binocular optic disc drusen (Figure 2).

## Discussion

In previous studies, PHOMS was found in cases of optic disc drusen (ODD), optic disc edema (ODE), pseudo-optic disc edema, tilted disc syndrome (TDS), and optic neuritis, as well as other diseases (8, 12–15). We found that PHOMS was also present in ischemic optic neuropathy and white dot syndromes. Further, some patients may be developed into macular neovascularization (MNV). Of the 26 patients (37 eyes) that we included in this study, 12 were male and 14 were female. The average age was 38.65 ± 18.76 (Mean ± SD) years. The average BCVA was 0.55 ± 0.38 (Mean ± SD). The average BCVA of patients under 50 years old was 0.54 ± 0.38 (Mean ± SD) and that of patients over 50 years old was 0.58 ± 0.37 (Mean ± SD). There was no significant difference, and there seemed to be no correlation between age and BCVA. Among them, including optic disc drusen (nine eyes), tilted disc syndrome (two eyes), optic neuritis (11 eyes), ischemic optic neuropathy (12 eyes), white dot syndromes (two eyes) and macular neovascularization (one eye). PHOMS widths ranged from 309 μm to 2285 μm (Mean ± SD: 721.14 ± 365.11). In females patients, there is only a 9-year-old girl whose PHOMS widths are larger. At the same time, there is also a 54-year-old man whose PHOMS width is larger. We think this was caused by individual differences. Excluding these two patients, the average PHOMS width of male patients was 629.67 ± 174.52 μm (Mean ± SD) and that of female patients was 627 ± 151.72 μm (Mean ± SD). There was no significant difference. Their heights ranged from 194 μm to 839 μm (Mean ± SD: 399.30 ± 145.98). We also found that PHOMS tended to be located outside and around the optic disc. PHOMS is defined as a hyperreflective ovoid mass-like structure with a hyperreflective interior without sharp outer edges or a hyporeflective core. PHOMS can typically not be found using fluorescence methods, and is not visible on B-scan ultrasonography despite its superficial location (16). It can also be seen in patients without apparent optic disc edema or poorly demarcated optic discs. During our follow-up, we found that PHOMS did not disappear. These findings are roughly the same as what has been previously reported in the literature (8, 9, 11, 17).

Early definitions of PHOMS were made with the context that they often occur alongside optic disc drusen (ODD) (18). With the development of multi-modal imaging, however, it appears that there are several features of PHOMS that are distinct from ODD, even though some symptoms overlap (8, 12, 13, 19–21). PHOMS does tend to be more common in patients with ODD. However, 26% of patients without ODD but with disc edema from NODD-AION had PHOMS (22). PHOMS do not correspond to clinically visible ODD. The same OCT findings can also be seen in patients without ODD (16).

In addition, PHOMS should be differentiated from true optic disc edema. Optic disc edema is caused by increased intracranial pressure or optic neuritis, and can be a serious threat to vision and life (3, 23). PHOMS may be caused by some degree of optic disc congestion or axonal stasis (17). Under certain circumstances, it is necessary to examine the patient's intracranial pressure at the time of initial diagnosis, and to examine the cause of intracranial pressure, using methods such as neuroimaging and lumbar punctures. In addition, OCT features of normal vessels are similar to those of vessels with PHOMS. However, the retina's vascular structures are located on the surface of the optic disc, and PHOMS structures are located subretinally and above Bruch's membrane (14). PHOMS were also observed in patients with myopic tilted discs, which is not related to papilledema nor ODD. It is now suggested, PHOMS are non-specific OCT findings of axonal distension and crowding that can be seen in acquired and dysplastic anomalies of the ONH (16). PHOMS is a new clinical feature found on the EDI-OCT in recent years. Multi-mode imaging has been proved to be a better way to identify PHOMS. EDI-OCT is the most sensitive and specific technique to identify PHOMS and thus serves as the mainstay of diagnosis.

Later with the advent of OCTA, we also found vascular complexes in PHOMS using OCTA. The OCTA revealed significant blood flow signals in these hyperreflective lesions, which may indicate there are vascular structures within them. This phenomenon has been described in several previous studies based on OCTA findings (8, 12, 13, 15), but PHOMS is a new clinical feature that has been demonstrated using EDI-OCT in recent years. Multi-modal imaging, especially OCTA, may be a better way to identify PHOMS.

Most researchers think PHOMS is caused by herniation of nerve fibers or axoplasmic stasis, or by prelaminar optic nerve head. Some histopathological evidence suggests that PHOMS is related to lateral bulges of nerve fibers in the retina. It might bring deeper vessels deputed at the irroration of the optic nerve into the retina. It may even lead to increased levels of vascular endothelial growth factor (VEGF) and the development of neovessels (8–10, 24), which could then lead to secondary CNV. Studies by Shinohara and Pichi et al. (based on OCT images) showed that the PHOMS protrudes from the upper edges of Bruch's membrane and choroid, and courses toward. This may help pinpoint PHOMS locations (12, 13). In recent years, OCTA technology has been able to better display fundus blood flow lesions, thus expanding the evaluation of deeper vascular layers (25). However the exact mechanisms that lead to the formation of PHOMS remain unknown, and more study is needed.

There are some limitations of our study. Patients' clinical features were not collected completely due to unfamiliarity with PHOMS features. This study is also limited to morphological analysis of PHOMS, and the exact pathophysiology remains unknown. In addition, due to the difficulty of following up on some patients, sufficient longitudinal data were not always collected. As a result, we have not carried out a comprehensive longitudinal discussion in this respect. Our study also had a small case number. We also did not look at the correlation between PHOMS and visual field defects. Although we have collected the quantitative measurement of PHOMS, certain clinical characteristics of PHOMS, and the pathogenesis of the disease, need further analysis. This could look like studying in whether PHOMS can be used to predict visual outcomes in neuro-ophthalmic disease, whether PHOMS size is associated with other measurements of optic disc edema, whether early discovery of PHOMS by OCT could be used to predict papilledema recurrence, and whether the size or location of a PHOMS could predict visual field (15). In order to improve the accuracy and robustness of the conclusions and provide better clinical guidance, we need to conduct more comprehensive research in the subsequent clinical work.

## Conclusion

EDI-OCT is the most sensitive and specific technique to identify PHOMS and thus serves as the mainstay of diagnosis. EDI-OCT of PHOMS showed hyperreflective structures surrounded by hyporeflective edges around the optic discs. These characteristics may be unique features revealed by EDI-OCT and can be found in a variety of optic nerve diseases. Later with the advent of OCTA, we found that it may be developed into CNV. Future larger longitudinal studies may reveal the exact mechanisms leading to the OCT finding.

## Data Availability Statement

The original contributions presented in the study are included in the article/supplementary material, further inquiries can be directed to the corresponding author.

## Ethics Statement

The study was approved by the Institutional Ethics Committee of Shandong Eye Hospital (approval No. 2019-G-012) on March 12, 2019. Written informed consent to participate in this study was provided by the participants' legal guardian/next of kin. Written informed consent was obtained from the individual(s), and minor(s)' legal guardian/next of kin, for the publication of any potentially identifiable images or data included in this article.

## Author Contributions

TL and XX designed research, conducted clinical examination, and drafted and revised the manuscript. WW, GT, JW, JG, MC, XW, and QZ generated the figures and tables and organized the data and tables. All authors contributed to the article and approved the submitted version.

## Funding

This study was supported by Natural Key Research and Development Project (2016YFC1305500).

## Conflict of Interest

The authors declare that the research was conducted in the absence of any commercial or financial relationships that could be construed as a potential conflict of interest.

## Publisher's Note

All claims expressed in this article are solely those of the authors and do not necessarily represent those of their affiliated organizations, or those of the publisher, the editors and the reviewers. Any product that may be evaluated in this article, or claim that may be made by its manufacturer, is not guaranteed or endorsed by the publisher.
